# Cardiac effects of two hallucinogenic natural products, *N*,*N*-dimethyl-tryptamine and 5-methoxy-*N*,*N*-dimethyl-tryptamine

**DOI:** 10.1038/s41598-025-91400-6

**Published:** 2025-02-25

**Authors:** Joachim Neumann, Tobias Dietrich, Karyna Azatsian, Britt Hofmann, Ulrich Gergs

**Affiliations:** 1 Institut für Pharmakologie und Toxikologie, Medizinische Fakultät, Martin-Luther-Universität Halle-Wittenberg, Magdeburger Str. 4, 06097 Halle, Germany; 2 Department of Pharmacology and Toxicology, Faculty of Pharmacy, Gdansk, Poland; 3 Department of Cardiac Surgery, Mid-German Heart Center, University Hospital Halle, 06097 Halle, Germany

**Keywords:** Serotonin, *N*,*N*-dimethyl-tryptamine (DMT), 5-Methoxy-*N*,*N*-dimethyl-tryptamine (5-MeO-DMT), 5-HT_4_ receptor, Inotropy, Chronotropy, Transgenic mice, Human atrium, Phospholamban, Drug safety, Receptor pharmacology, Adverse effects, Depression

## Abstract

It is unclear whether hallucinogenic tryptamine derivatives namely *N*,*N*-dimethyl-tryptamine (DMT) and 5-methoxy-*N*,*N*-dimethyl-tryptamine (5-MeO-DMT) exert positive inotropic effects in the human heart. Therefore, we measured the inotropic effects of these drugs in isolated left and right atrial preparations of mice that overexpress human 5-HT_4_ receptors (5-HT_4_-TG) and preparations from wild type mice (WT). Moreover, we measured force of contraction in isolated right atrial preparations from adult patients, obtained in the process of open heart surgery due to severe coronary heart disease. DMT and 5-MeO-DMT augmented the force of contraction in isolated paced (1 Hz) left atrial preparations from 5-HT_4_-TG and raised the spontaneous beating rate of right atrial preparations from 5-HT_4_-TG. The drugs elevated force of contraction in paced (1 Hz) human right atrial muscle preparations. The maximum inotropic effects of DMT and 5-MeO-DMT were smaller at 10 µM (about 65%) than that of 1 µM 5-HT on the left atria from 5-HT_4_-TG. The maximum increase in the beating rate due to DMT and 5-MeO-DMT amounted 40 ± 5% of the effect of 5-HT on right atrial preparations from 5-HT_4_-TG (*n* = 5–6). DMT and 5-MeO-DMT were inactive in WT. The potency of 5-MeO-DMT to increase force of contraction could be increased by pre-treatment of human atrial preparations by the phosphodiesterase inhibitor cilostamide (1 µM). 5-MeO-DMT increased the phosphorylation state of phospholamban at serine 16 in isolated left atrial muscle strips of 5-HT_4_-TG. In summary, DMT and 5-MeO-DMT acted as partial agonists on human 5-HT_4_ receptors.

**Supplementary Information:**

The online version contains supplementary material available at 10.1038/s41598-025-91400-6.

## Introduction

**Fig. 1:**
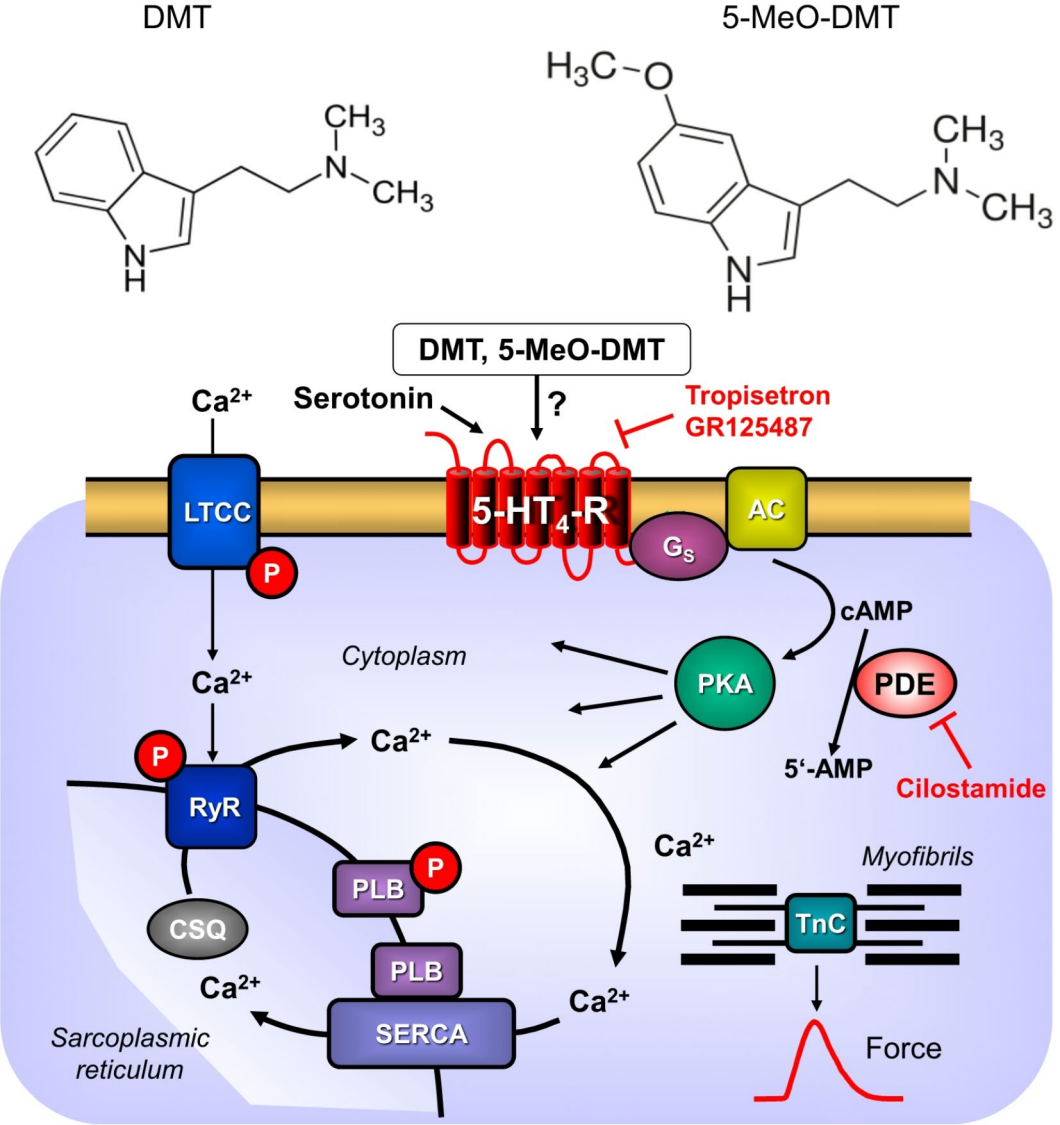
Structural formulas and proposed mechanism of action of DMT and 5-MeO-DMT. Ca^2+^ passes the cardiac myocyte membrane via the L-type Ca^2+^ channel (LTCC). This process can be enhanced by serotonin (5-HT), DMT and 5-MeO-DMT via activation of the 5-HT_4_ receptor (inhibitable for example by tropisetron or GR125487). Agonists binding elevate the activity of the adenylyl cyclase (AC) in the sarcolemma via stimulatory G-proteins (G_s_). This elevates the cytosolic cAMP followed by activation of the cAMP-dependent protein kinase (PKA). PKA increases the phosphorylation state (P) of many proteins including LTCC, phospholamban (PLB) and the inhibitory subunit of troponin (TnI). These phosphorylation events lead to increased cardiac force generation and relaxation. When Ca^2+^ passes through the LTCC, it can get into the vicinity of the ryanodine receptors (RYR). This leads finally to opening of the RYR and more Ca^2+^ flows into the cytosol of the cardiomyocyte. When this released Ca^2+^ binds to myofilaments, they contract more vigorously and this leads to an increase in force of contraction. In diastole, Ca^2+^ is pumped into the sarcoplasmic reticulum via a sarcoplasmic reticulum Ca^2+^ ATPase (SERCA). The activity of SERCA is enhanced by an augmented phosphorylation state of PLB.

The natural occurring molecules 5-methoxy-*N*,*N*-dimethyl-tryptamine (5-MeO-DMT) and *N*,*N*-dimethyl-tryptamine (DMT) are hallucinogenic drugs. They occur in South America mainly in plants of the Amazonas region^1,2^. Perorally given alone, 5-MeO-DMT is inactivated rapidly by a first pass effect^3^. Hence, 5-MeO-DMT has to be injected or has to be supplemented with inhibitors of the enzymatic degradation^3^. These enzyme inhibitors could be antidepressant drugs like tranylcypromine. There are also reports in the literature that pure 5-MeO-DMT was mixed with plants extracts harmine or harmaline to inhibit the first pass effect^4^. DMT and 5-MeO-DMT are chemically similar to serotonin (5-HT; 5-hydroxy-tryptamine) and therefore can bind to several seven known classes of serotonin receptors. Stimulation of brain 5-HT_2A_-receptors explains the hallucinogenic effects of DMT and 5-MeO-DMT^5^. DMT but not 5-MeO-DMT increased the beating rate in isolated rabbit hearts^6^. However, serotonin acts in rabbit hearts by release of noradrenaline and not via serotonin receptors and hence rabbit hearts are not a good model for the human heart^7^. In contrast to rabbits, in rats, 5-MeO-DMT decreased the heart rate^8^. These effects were suggested to be due to stimulation of 5-HT_1_ serotonin receptors^8^. We had reported, in contrast, that 5-HT increased force of contraction in isolated atrial preparations from rats via 5-HT_2A_ serotonin receptors^9^. However, as far as we know, inotropic effects of DMT and 5-MeO-DMT have not yet been reported via 5-HT_4_ receptors from any species. We have chosen to study these tryptamine derivatives because they are naturally occurring hallucinogenic drugs, forbidden to use in many countries, but popular for “recreational” purposes and sometimes leading to fatal intoxications. Thus, from a clinical perspective it would be helpful to know whether or not these drugs act as agonists at cardiac 5-HT_4_ receptors, because then 5-HT_4_ receptor antagonists could be used to treat the cardiac side effects of these intoxications. Hence, we tested the hypothesis that DMT and 5-MeO-DMT act as agonists on cardiac human 5-HT_4_ serotonin receptors (Fig. 1). Parts of the data have been reported in abstract form^10,11^.

## Materials and methods

### Transgenic mice

A mouse with cardiomyocyte-specific expression of the human 5-HT_4a_ receptor has been generated in our laboratories^12^. The cardiac myocyte-specific expression was achieved by the use of the α-myosin heavy chain promoter. The age of the animals studied in the atrial contraction experiments was around 154 days. All mice were housed under conditions of optimum light, temperature and humidity with food and water provided ad libitum. The investigation conformed to the Guide for the Care and Use of Laboratory Animals as published by the National Research Council (2011). The animals were handled and maintained according to the approved protocols of the Animal Welfare Committee of the University of Halle-Wittenberg, Halle, Germany. The study was conducted in accordance with ARRIVE guidelines^13^.

### Contractile studies on mouse atrial preparations

In brief, mice were euthanized by intraperitoneal injection of sodium pentobarbital (250 mg/kg body weight)^14^. Then, the right and left atrial preparations were isolated and mounted in organ baths as previously described^15,16^. The bathing solution of the organ baths contained 119.8 mM NaCI, 5.4 mM KCI, 1.8 mM CaCl_2_, 1.05 mM MgCl_2_, 0.42 mM NaH_2_PO_4_, 22.6 mM NaHCO_3_, 0.05 mM Na_2_EDTA, 0.28 mM ascorbic acid and 5.05 mM glucose. The solution was continuously gassed with 95% O_2_ and 5% CO_2_ and maintained at 37 °C and pH 7.4^16–18^. Spontaneously beating right atrial preparations from mice were used to study any chronotropic effects and the left atrial preparations were field stimulated with a frequency of 1 Hz to study force of contraction.

The drug application was as follows. After equilibration was reached, 1 nM to 10 µM DMT or 5-MeO-DMT was added to the atrial preparations to establish concentration-response curves followed directly by a concentration-response curve of 5-HT (1 nM to 1 µM). This was to test if DMT or 5-MeO-DMT behave as full or partial agonists. After washout, again a concentration-response curve for DMT or 5-MeO-DMT (1 nM to 10 µM was performed to test if there are any desensitization effects that could compromise the results.

### Contractile studies on human atrial preparations

The contractile studies on human preparations were done using the same setup and buffer as used in the mouse studies (see section above). The right atrial preparations were obtained from 14 male and two female patients aged 59–78 years (mean ± SD: 68.9 ± 6.4 years) undergoing bypass surgery. Further details on patient characteristics are summarized in Table 1. Our methods used for atrial contraction studies in human samples have been previously published and were not altered in this study^19–22^. This study has been performed in accordance with the Declaration of Helsinki. The study protocol was approved by the local ethics committee of the Medical Faculty of the Martin Luther University Halle-Wittenberg (Ethics approval number: hm-bü 04.08.2005) and all research was performed in accordance with relevant guidelines/regulations. Informed consent was obtained from all patients included in the study.

**Table 1:** Patient characteristics.

Patient ID	Sex	Age (years)	NYHA class	CCS angina grading scale	LVEF (%)	Cardiac catheterization findings	Medication
#1	M	78	III	IV	65	2 vessel CHD	Acetylsalicylic acid, ramipril, metoprolol, torasemide, pantoprazole, alendronic acid, methylprednisolone, insulin
#2	F	64	III–IV	III–IV	35	2 vessel CHD, AoS	Acetylsalicylic acid, atorvastatin, telmisartan, hydrochlorothiazide, pantoprazole, anastrozole, metformin
#3	M	74	II	III–IV	60	2 vessel CHD, NSTEMI	Metoprolol, apixaban, atorvastatin, torasemide
#4	M	74	III	IV	40	2 vessel CHD	Acetylsalicylic acid, bisoprolol, atorvastatin, ramipril, prasugrel, dapagliflozin, sitagliptin, pantoprazole, torasemide
#5	M	74	II	III–IV	55	3 vessel CHD, STEMI	Acetylsalicylic acid, bisoprolol, atorvastatin, clopidogrel, torasemide, valsartan
#6	M	59	III	III	40	3 vessel CHD, NSTEMI	Acetylsalicylic acid, metoprolol, allopurinol, clopidogrel, pantoprazole, simvastatin, torasemide
#7	M	77	II	III	55	3 vessel CHD, LMCA stenosis	Clopidogrel, apixaban, nebivolol, calcium acetate, doxazosin, moxonidine, pantoprazole, sevelamer carbonate, torasemide, methylprednisolone, xipamide
#8	M	61	III	III	72	3 vessel CHD, LMCA stenosis	Acetylsalicylic acid, bisoprolol, allopurinol, atorvastatin, enalapril, hydrochlorothiazide, omega-3-acid ethyl esters, pantoprazole, budesonide, salbutamol
#9	M	72	III	III–IV	55	2 vessel CHD, NSTEMI	Acetylsalicylic acid, torasemide, pantoprazole, tamsulosin, ezetimibe, candesartan, atorvastatin, amlodipine, apixaban
#10	M	71	II–III	III	50	3 vessel CHD, AoS	Acetylsalicylic acid, metoprolol, amlodipine, atorvastatin, chlorthalidone, moxonidine, pantoprazole, torasemide, valsartan, insulin
#11	M	60	II	III	50	3 vessel CHD, LMCA stenosis	Acetylsalicylic acid, metoprolol, candesartan, dapagliflozin, sitagliptin, rosuvastatin, metformin, clopidogrel, terbinafine
#12	M	59	III	III	30	3 vessel CHD, ICM	Acetylsalicylic acid, metoprolol, atorvastatin, sacubitril/valsartan, ezetimibe, dapagliflozin, metformin, pantoprazole, spironolactone, torasemide
#13	F	75	III	III–IV	40	3 vessel CHD, NSTEMI, ICM	Acetylsalicylic acid, metoprolol, atorvastatin, ezetimibe, ramipril, pantoprazole, prednisolone, torasemide, metamizole
#14	M	71	III	III–IV	55	3 vessel CHD, STEMI, AoS	Acetylsalicylic acid, metoprolol, atorvastatin, candesartan, ezetimibe, pantoprazole, torasemide
#15	M	68	III–IV	III	23	3 vessel CHD, NSTEMI, AoS	Acetylsalicylic acid, metoprolol, amlodipine, atorvastatin, sacubitril/valsartan, eplerenone, glimepiride, dapagliflozin, pantoprazole, metformin, budesonide/formoterol, torasemide, insulin
#16	M	65	III	III	40	3 vessel CHD, LMCA stenosis, STEMI	Acetylsalicylic acid, metoprolol, acetyl cysteine, atorvastatin, levothyroxine, pantoprazole, torasemide, metamizole, insulin
Mean ± SD		68.9 ± 6.4			47.8 ± 12.7		

*NYHA* New York Heart Association, *CCS* Canadian Cardiovascular Society, *LVEF* left ventricular ejection fraction, *CHD* coronary heart disease, *AoS* aortic stenosis, *STEMI* ST-segment elevation myocardial infarction, *NSTEMI* non-ST-segment elevation myocardial infarction, *LMCA* left main coronary artery, *ICM* ischemic cardiomyopathy.

### Western blotting

The homogenization of the samples, protein measurements, electrophoresis, primary and secondary antibody incubation and quantification were performed following our previously established protocols^19,23–25^. Briefly, samples were homogenized in a buffer containing 10 mM NaHCO_3_ and 5% SDS. Electrophoresis was performed in Novex™ 4–20% “Tris–Glycine Plus Midi Protein Gels” (Invitrogen, Thermo Fisher Scientific, Waltham, Massachusetts, USA). Subsequently, the proteins were transferred to a nitrocellulose membrane (Amersham Protran 0.45 µM, Cytiva, Germany) by wet transfer in a phosphate buffer (42 mM Na_2_HPO_4_, 8 mM NaH_2_PO_4_) for four Ampere hours at 4 °C. Following primary antibodies were used: anti serine 16-phosphorylated phospholamban (PS16-PLB; 1:5000; #A010-12AP; Badrilla, Leeds, UK), anti calsequestrin as a cardiac myocytes-specific loading control (CSQ; 1:20.000; #ab3516; abcam, Cambridge, UK). The signals were visualized by using chemiluminescence (Immobilon™ Western, Millipore, Merck; Darmstadt, Germany) and a digital imaging system (Amersham ImageQuant 800; Cytiva Europe GmbH, Freiburg im Breisgau, Germany).

### Data analysis

Data shown are means ± standard deviation. Recordings and primary analyses of contraction data were done with LabChart 8 (ADInstruments, Spechbach, Germany) and primary analyses of Western blots were performed with ImageQuant 10 (Cytiva, Freiburg, Germany). Statistical analyses and preparation of graphics were done with Prism 9.0 (GraphPad Software, San Diego, California, USA) using the analysis of variance followed by Bonferroni’s posttest. A *p* value < 0.05 was considered to be significant.

### Drugs and materials

Serotonin (5-HT) hydrochloride was purchased from Sigma-Aldrich (Germany). Dimethyl-tryptamine (DMT) was purchased as solution (1 mg/ml in methanol) from Sigma-Aldrich (Germany) or as solid compound from LGC GmbH (Luckenwalde, Germany). 5-methoxy-dimethyl-tryptamine (5-MeO-DMT) was purchased as solid compound from Sigma-Aldrich (Germany) as well as from LGC GmbH (Luckenwalde, Germany). Both drugs were diluted/dissolved in a 50% DMSO and 50% water mixture and stored at − 20 °C. GR125487 was purchased from TOCRIS (Bio-Techne, Wiesbaden, Germany). All other chemicals were of the highest purity grade commercially available. Deionized water was used throughout the experiments. Stock solutions were prepared fresh daily.

## Results

### Studies in the isolated left atria from mice

We have previously shown that 5-HT increases the force of contraction in left atria from 5-HT_4_-TG, but not in left atria from WT^15^. Here, as a next step, we wanted to compare those data with those of DMT and 5-MeO-DMT, and we wanted to determine whether they also exert positive inotropic effects in 5-HT_4_-TG.

**Fig. 2:**
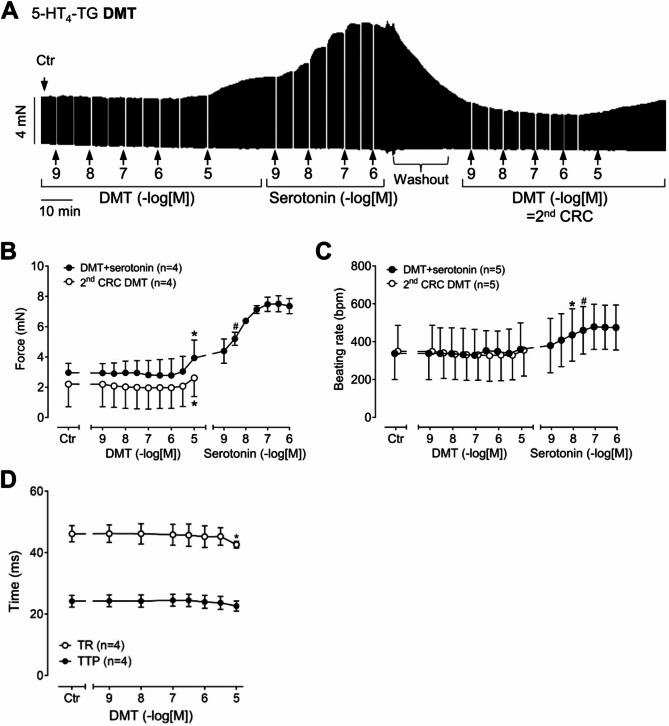
Effects of DMT on mouse atrial preparations. (**A**) Original recording: Effect of DMT and serotonin on force of contraction in an isolated paced (1 Hz) left atrial preparation of a 5-HT_4_ receptor overexpressing mouse (5-HT_4_-TG). At the end of the concentration response curve for DMT (CRC), serotonin (5-HT) was applied. Then washout was performed and again DMT was given (2nd CRC). Horizontal bar: time axis in minutes (min). Vertical bar: developed tension in milli Newton (mN). (**B**) Summarized effects of DMT and serotonin on force of contraction in isolated electrically driven (1 Hz) left atria of 5-HT_4_-TG mice. (**C**) Summarized effects of DMT and serotonin on the beating rate in isolated spontaneously beating right atria of 5-HT_4_-TG mice. (**D**) Summarized effects of DMT on time to peak tension (TTP) and time of relaxation (TR) in left atria of 5-HT_4_-TG mice. *First significant difference (*p* < 0.05) versus control (Ctr, predrug value); ^#^First significant difference (*p* < 0.05) versus 10 µM DMT. Numbers in brackets indicate number of experiments.

Like serotonin also its derivative DMT (original recording: Fig. 2A) raised force in a concentration- and time-dependent manner in left atrial preparations from 5-HT_4_-TG. The data on force of contraction are summarized in Fig. 2B and the time parameters of the contraction are summarized in Fig. 2D. Corresponding to the increase of the force at 10 µM DMT, the time of relaxation was shortened, indicative for a cAMP-dependent mechanism. Thereafter, we applied additionally in a cumulative way increasing concentrations of 5-HT (Fig. 2A). DMT was less potent and less effective than 5-HT (pEC_50_ = 8.3) to raise force of contraction (Fig. 2A,B). Previously, we had noted that 5-HT rapidly and effectively desensitized the 5-HT_4_ receptor under our experimental conditions^26^. Hence, the question arose whether DMT would also lead to functional desensitization. Therefore, we washed out the effects of DMT and 5-HT (Fig. 2A, washout), and subsequently, DMT was reapplied cumulatively. Once more, DMT elicited a positive inotropic effect (Fig. 2A). These data are summarized in Fig. 2B (second CRC). In parallel, by using the spontaneously beating right atria, we estimated whether under these conditions DMT affected the beating rate. As depicted in Fig. 2C, DMT exerted a very small, negligible positive chronotropic effect. Like in Fig. 2A, we then added cumulatively 5-HT. DMT was less potent and less effective to raise beating rate than 5-HT (Fig. 2C). DMT, like 5-HT^15^, failed to affect the force of contraction or the beating rate in WT (data not shown).

**Fig. 3:**
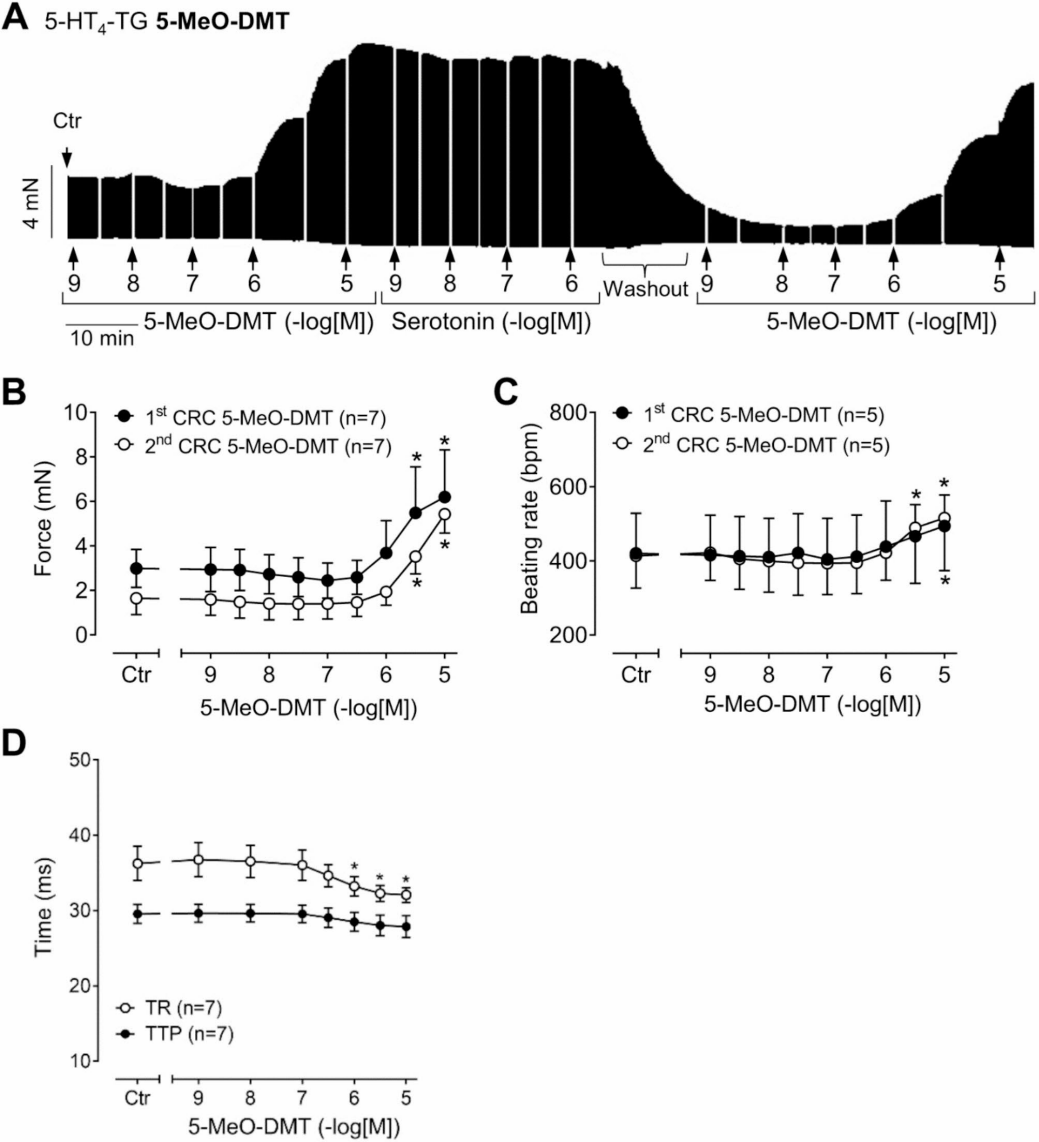
Effects of 5-MeO-DMT on mouse atrial preparations. (**A**) Original recording: Effect of 5-MeO-DMT and serotonin on force of contraction in an isolated paced (1 Hz) left atrial preparation of a 5-HT_4_ receptor overexpressing mouse (5-HT_4_-TG). At the end of the concentration response curve for 5-MeO-DMT (1st CRC), serotonin (5-HT) was applied. Then washout was performed and again 5-MeO-DMT was given (2nd CRC). Horizontal bar: time axis in minutes (min). Vertical bar: developed tension in milli Newton (mN). (**B**) Summarized effects of 5-MeO-DMT on force of contraction in isolated electrically driven (1 Hz) left atria of 5-HT_4_-TG mice. (**C**) Summarized effects of 5-MeO-DMT on the beating rate in isolated spontaneously beating right atria of 5-HT_4_-TG mice. (**D**) Summarized effects of 5-MeO-DMT on time to peak tension (TTP) and time of relaxation (TR) in left atria of 5-HT_4_-TG mice. *p < 0.05 versus control (Ctr, predrug value). Numbers in brackets indicate number of experiments.

Next, we tested 5-MeO-DMT and found that 5-MeO-DMT raised force in a concentration- and time-dependent manner in left atrial preparations from 5-HT_4_-TG (original recording: Fig. 3A). The data on force of contraction are summarized in Fig. 3B and the time parameters of the contraction are summarized in Fig. 3D. Compared to DMT, 5-MeO-DMT was more potent and effective to raise force of contraction and, accordingly, the shorting of time parameters was more pronounced (Fig. 3D). Thereafter, as done for DMT, we applied additionally in a cumulative way increasing concentrations of 5-HT (Fig. 3A). 5-MeO-DMT (pEC_50_ ~ 5.8) seemed to be less potent but as effective as 5-HT to raise force of contraction (Fig. 3A). In other words, additionally applied 5-HT could not raise force of contraction further (Fig. 3A). However, given the experimental setup, it was not clearly possible to determine whether 5-MeO-DMT is a partial agonist in 5-HT_4_-TG, as the preparations had reached their maximal ability concerning contraction and beating rate. Therefore, the serotonin component was omitted from the graph of Fig. 3B. Finally, after washout, 5-MeO-DMT was applied again and 5-MeO-DMT induced again a positive inotropic effect (Fig. 3A). These data are summarized in Fig. 3B (second CRC). Here, also the right atria were used to study whether under these conditions 5-MeO-DMT affected the beating rate. As depicted in Fig. 3C and 5-MeO-DMT exerted a positive chronotropic effect. Like in left atria, we then added cumulatively 5-HT. 5-MeO-DMT seemed to be less potent but as effective as 5-HT to raise the beating rate, but for the reasons described above, the serotonin component was omitted from the graph of Fig. 3C. 5-MeO-DMT, like 5-HT^15^, failed to affect the force of contraction or the beating rate in WT (data not shown).

**Fig. 4:**
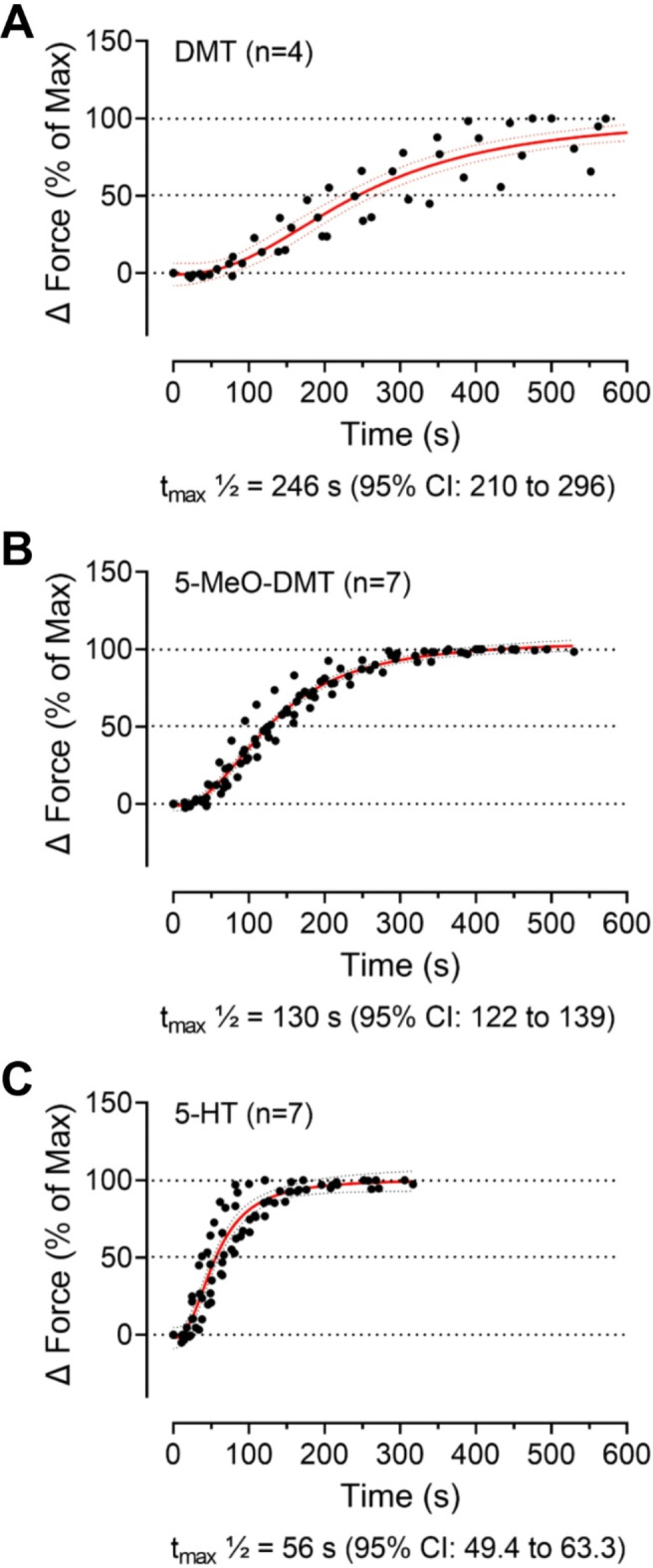
Time-dependent increase of force of contraction in isolated electrically stimulated (1 Hz) left atria of 5-HT_4_-TG mice. (**A**) Time-dependent effect of 10 µM DMT, (**B**) of 3 µM 5-MeO-DMT, and (**C**) of 10 nM serotonin (5-HT). At the specified drug concentrations, the developed force of contraction was expressed as delta force normalized to the maximum effect. The time to reach the half maximum effect (t_max_½) is provided below the corresponding graph, along with the 95% confidence interval (CI). *p < 0.05 versus control (Ctr, predrug value). Numbers in brackets indicate number of experiments.

Even though we could not calculate the EC_50_ values for DMT and 5-MeO-DMT because the concentration-response curves did not reach a plateau in the concentration range achievable in this study, it is obvious that 5-HT is the most potent compound, followed by 5-MeO-DMT, while DMT has the lowest potency. This order is reflected in the kinetic parameters of the substances, as shown in Fig. 4. The time to reach the maximum force of contraction was shortest for 5-HT and slowest for DMT (Fig. 4). In detail, 5-HT (t_max50_ = 56 s) increased the force of contraction approximately twice as fast as 5-MeO-DMT (t_max50_ = 130 s) and 5-MeO-DMT increased the force of contraction approximately twice as fast as DMT (t_max50_ = 246 s) (Fig. 4).

**Fig. 5:**
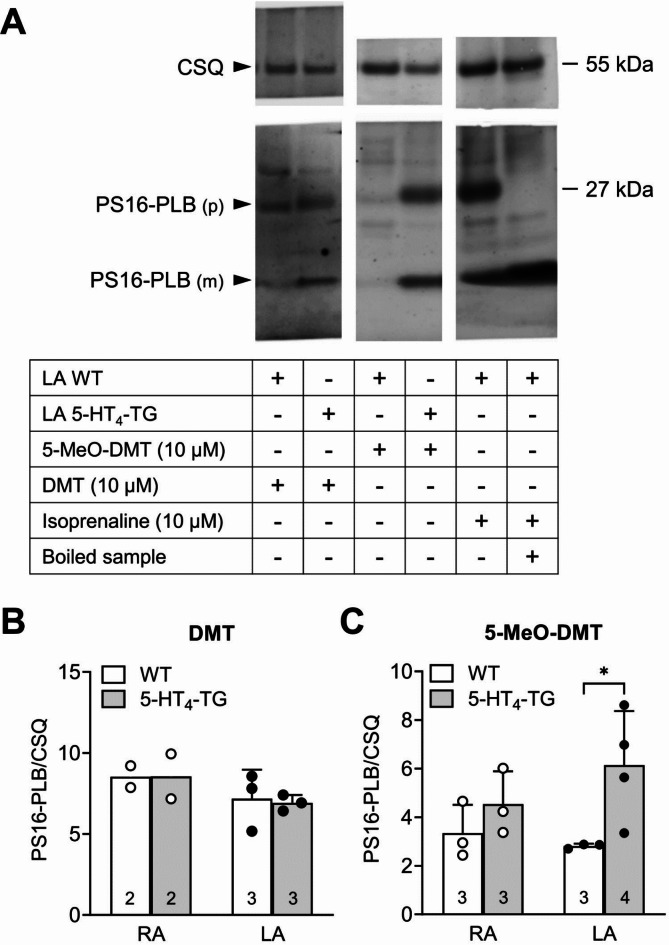
Phosphorylation of phospholamban (PLB) in mouse atrial preparations. (**A**) Typical Western blots: Effect of 10 µM 5-MeO-DMT and DMT on PLB serine-16 phosphorylation (PS16-PLB) in isolated electrically stimulated left atrium (LA) from wild type (WT) and 5-HT_4_ transgenic (5-HT_4_-TG) mice. Isoprenaline was used as positive control and boiling of the control sample shifted the PLB band from the pentameric form (p) to the monomeric form (m): this effect was used to identify the PLB band in the Western blot. Calsequestrin (CSQ) a constitutively expressed cardiac protein was used as loading control. For assessment of the expression of different proteins on one gel, the blots were cut and the lower and upper halves were incubated with different primary antibodies. (**B**) Summarized effects of DMT on the phosphorylation state of PLB in left atria (LA) and right atria (RA) of WT and 5-HT_4_-TG mice. (**C**) Summarized effects of 5-MeO-DMT on the phosphorylation state of PLB in LA and RA of WT and 5-HT_4_-TG mice. *p < 0.05 versus WT. Numbers in bars indicate number of experiments.

As depicted in the scheme in Fig. 1, we hypothesized that DMT and 5-MeO-DMT would increase the phosphorylation state of phospholamban at serine-16 (PS16-PLB). Hence, a separate set of contraction experiments was performed. We added 10 µM DMT or 5-MeO-DMT to left atrial preparations of 5-HT_4_-TG and WT until the maximum effect was reached (10 min) and then froze the atria. From these frozen atria, we performed and quantified Western blots. We noted that 5-MeO-DMT, but not DMT, increased the phosphorylation state of phospholamban in left atrial preparations from 5-HT_4_-TG but not WT (Fig. 5). This is depicted in original Western blots (Fig. 5A) and summarized in bar diagrams (Fig. 5B,C). A stimulation of β-adrenoceptors by isoprenaline was used as positive control. Moreover, boiling of the sample shifted the phospholamban band from the pentameric form to the monomeric form: this effect was used to clearly identify the phospholamban band in the Western blot (Fig. 5A).

### Studies in the isolated atria from humans

**Fig. 6:**
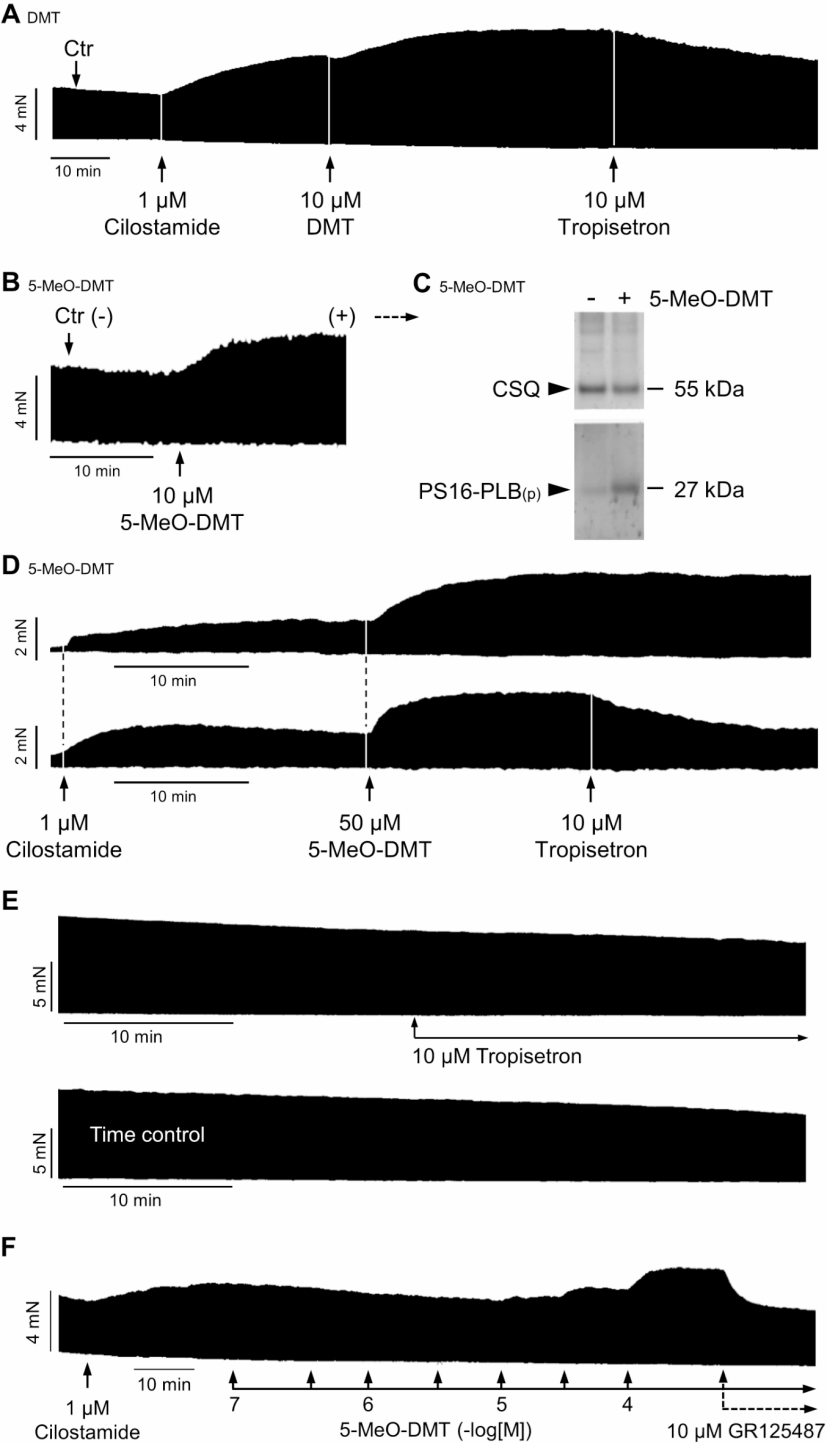
Effects of DMT and 5-MeO-DMT on isolated electrically driven (1 Hz) human right atrial preparations. (**A**) Effect of DMT on force of contraction in human right atrium. DMT alone was ineffective, but in the additional presence of cilostamide, a phosphodiesterase III inhibitor, a positive inotropic effect was seen that was antagonized by tropisetron. (**B**) Example of a patient where 5-MeO-DMT alone increased force of contraction and this increase was accompanied by an increased phosphorylation state at serine-16 of phospholamban (PS16-PLB) (**C**). Calsequestrin (CSQ) was used as loading control. (**D**) In other patients, 5-MeO-DMT increased force of contraction only in the presence of cilostamide and this positive inotropic effect of 5-MeO-DMT was antagonized by tropisetron. (**E**) Control experiments were performed with 10 µM tropisetron alone and in the absence of any compound (time control). (**F**) The positive inotropic effect of 5-MeO-DMT (in presence of cilostamide) was also antagonized by the specific 5-HT_4_ receptor antagonist GR125487. Horizontal bar in original recordings: time axis in minutes (min). Vertical bar in original recordings: developed tension in milli Newton (mN).

**Fig. 7:**
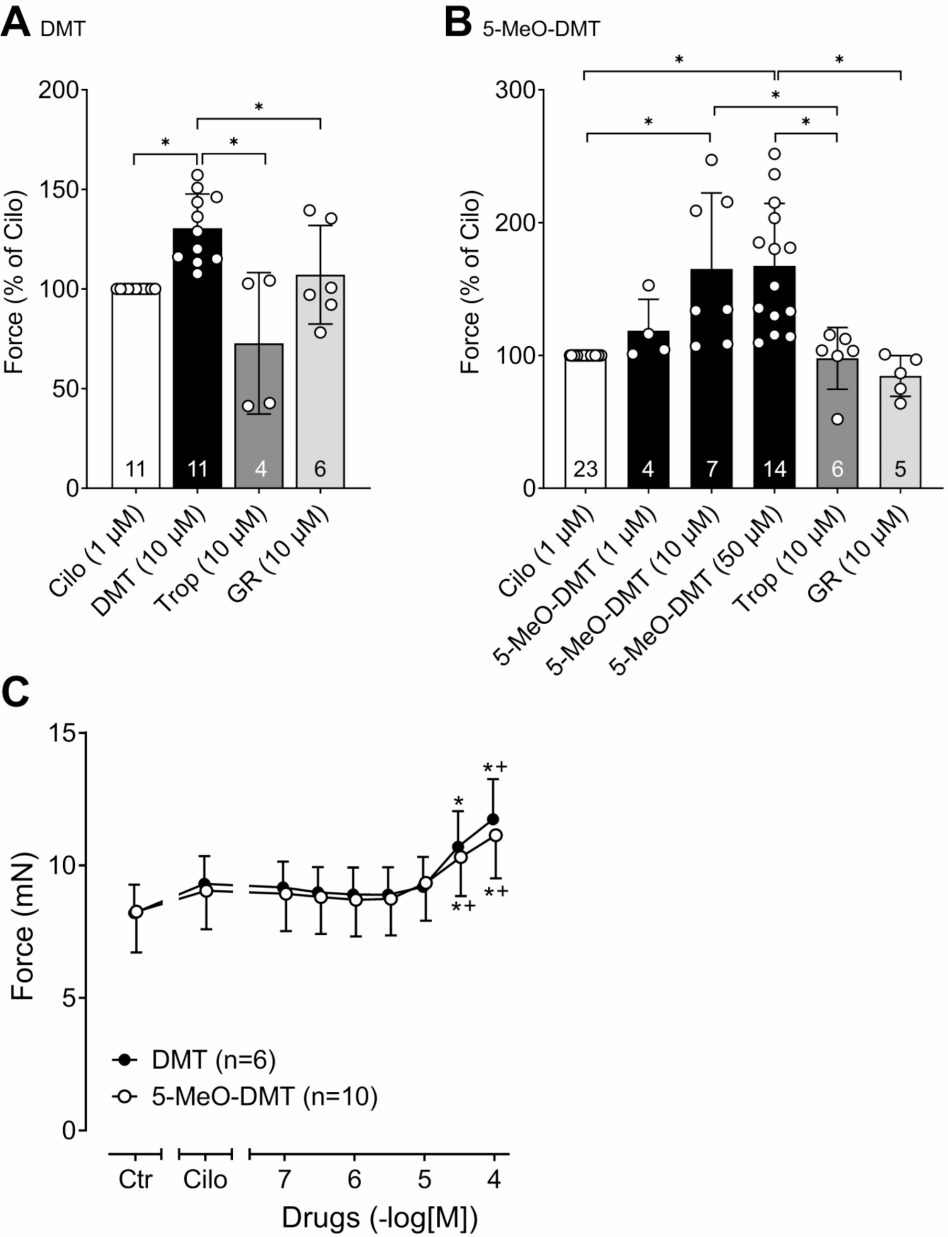
Summarized effects of DMT, DMT + tropisetron (Trop) and DMT + GR125487 (GR) (**A**) and 5-MeO-DMT, 5-MeO-DMT + tropisetron and 5-MeO-DMT + GR125487 (**B**) on force of contraction in human right atrium normalized to cilostamide (Cilo). *Significant differences (*p* < 0.05) between indicated groups. Numbers in bars indicate number of experiments. (**C**) concentration-response curves of DMT and 5-MeO-DMT in human right atrial preparations pre-stimulated with cilostamide (1 µM). *p < 0.05 versus control (Ctr, pre-drug value); ^+^*p* < 0.05 versus cilostamide. Numbers in brackets indicate number of experiments.

Now, the question arose whether these functional effects are confined to transgenic mice or also have clinical relevance in humans. Hence, we studied human atrial preparations to measure force under electrically stimulated isometric conditions. In general, the contraction data from human preparations showed a larger scatter compared to mouse preparations, which is due to the heterogeneity regarding, e.g., age, genetic background, health status, disease and medication of the patients included in the study (Table 1), which probably also applies to the serotonin receptor density. DMT alone did not increase force of contraction in isolated electrically paced right atrial muscle strips from patients (*n* = 5), but in the presence of the phosphodiesterase III inhibitor cilostamide, a positive inotropic effect of 10 µM DMT was seen (Fig. 6A). This positive inotropic effect of DMT was antagonized by tropisetron (Fig. 6A) and by the 5-HT_4_ receptor antagonist GR125487. The data are summarized in Fig. 7A. Tropisetron itself did not affect the force of contraction as demonstrated by the control experiments shown in Fig. 6E. The decline of force of contraction after 10 µM tropisetron was not different from the time-dependent decline of force of contraction (Fig. 6E). These control experiments were repeated three times, giving the same results.

**Fig. 8:**
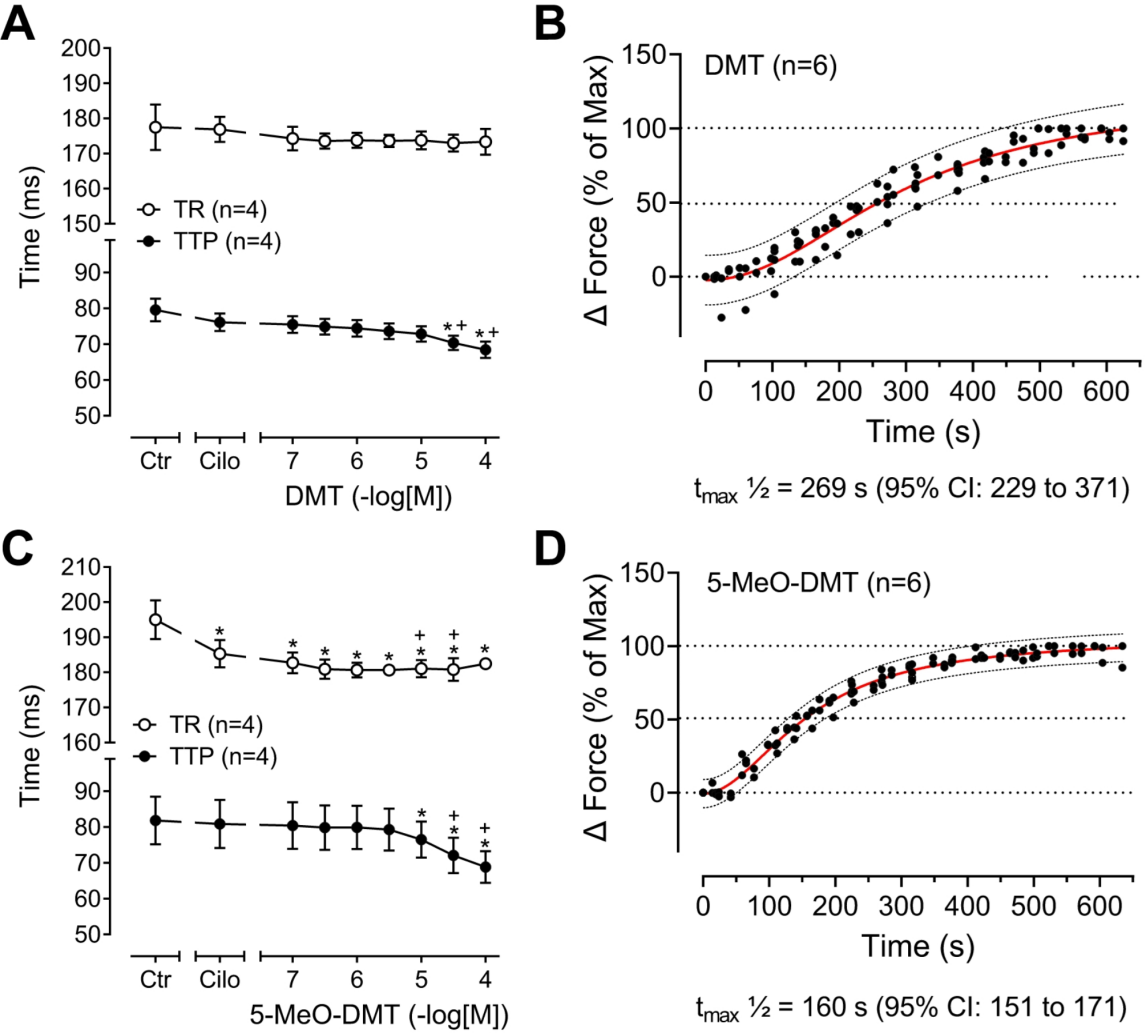
Effects of DMT (**A**) and 5-MeO-DMT (**C**) on time to peak tension (TTP) and time of relaxation (TR) in human right atrial preparations pre-stimulated with cilostamide (1 µM). (**B**) Time-dependent effect of 10 µM DMT and (**D**) of 10 µM 5-MeO-DMT. At the specified drug concentrations, the developed force of contraction was expressed as delta force normalized to the maximum effect. The time to reach the half maximum effect (t_max_½) is provided below the corresponding graph, along with the 95% confidence interval (CI). *p < 0.05 versus control (Ctr, predrug value); ^+^*p* < 0.05 versus cilostamide. Numbers in brackets indicate number of experiments.

The effects of 5-MeO-DMT were different from those of DMT. In the atrial preparations from some patients (*N* = 3) with an apparently high responsiveness to positive inotropic substances for unknown reasons, we noted that 5-MeO-DMT alone increased the force of contraction (Fig. 6B) and this increase was accompanied by an increased phospholamban phosphorylation (Fig. 6C). In the atrial preparations from other patients (*N* = 9) with an apparently lower responsiveness to positive inotropic substances, 5-MeO-DMT increased the force of contraction, as observed for DMT, only in the presence of cilostamide (Fig. 6D). This positive inotropic effect of 10 µM 5-MeO-DMT was antagonized by tropisetron (Fig. 6D) as well as by the 5-HT_4_ receptor antagonist GR125487 (Fig. 6F). The effect of 5-MeO-DMT was concentration-dependent und is summarized in Fig. 7B. In a further series of experiments, in the presence of cilostamide, concentration-response curves for DMT and 5-MeO-DMT were performed from 0.1 to 100 µM (Fig. 7C). Here, the potency and efficacy of DMT and 5-MeO-DMT appeared to be the same. Unfortunately, the pEC_50_ values could not be calculated accurately because the plateau of the concentration-response curves was not reached even at 100 µM, a concentration usually not reached in humans. An approximate estimate gave a pEC_50_ ≤ 4.5 for both DMT and 5-MeO-DMT in the human atrium. The evaluation of the time parameters revealed that the time to peak tension and the time of relaxation were shortened in a concentration-dependent manner by DMT and 5-MeO-DMT (Fig. 8A,C). The contraction kinetics of DMT and 5-MeO-DMT in the human atria (Fig. 8B,D) were similar to the kinetics found in the 5-HT_4_-TG atria (Fig. 4). That is, 5-MeO-DMT reaches the maximum inotropic effect for a given concentration almost twice as fast as DMT.

## Discussion

The new finding of this study is the observation that DMT, and particularly 5-MeO-DMT, increases cardiac contractility via human 5-HT_4_ receptors in the heart.

DMT occurs in plants^27^ and one has used DMT in religious settings^28,29^. DMT can be found, for instance, in leaves of *Diplopterys cabreana* in Colombia and Ecuador^30,31^. There are drug preparations in Brazil (Amazonas region) that are called ayahuasca: they include parts from the plant *Banisteriopsis caapi*. DMT is degraded by the enzyme monoamine oxidase A (MAO-A) that physiologically occurs in the gastrointestinal tract: therefore users added plant extracts that contain MAO-A inhibitors, which also inhibit MAO-B at higher concentrations^28,32^. Moreover, DMT is present in about 50 plants in South America^31^. The so called ayahuasca (a Quechua word translated as “vine of the souls”^31^) is a mixture of at least DMT and endogenous MAO inhibitors^28^. In more detail, ayahuasca is said to be prepared by combining the bark of the plant *Banisteriopsis caapi* vine and the leaves of the *Psychotria viridis* bush^29,31^. This mixture is boiled for hours and then swallowed since pre-Columbian times by the indigenous tribes of the Amazon Basin^29^. In Brazil, ayahuasca is used also for medical therapeutic purposes^31^. In mice, the lethal dose of DMT is about 47 mg/kg when given intraperitoneally^29^. From rodent studies, the LD_50_ of DMT in humans was calculated as 1.6 mg/kg if applied intravenously^29^. No human deaths have been reported due to ayahuasca, but when polypharmacy is involved and also 5-MeO-DMT has been taken, one death is reported in the literature^33^.

When DMT alone was administered by injection in humans (0.7–1.1 mg/kg body weight) they reported visual hallucinations^34^. In humans, a placebo controlled study with intravenous application of DMT led to peak DMT plasma concentrations of about 0.38 µM, an increase in heart rate, and an increase in blood pressure^35^. In our study, DMT hardly increased the beating rate in 5-HT_4_-TG mouse right atrial preparations, and the inotropic effects in human atria only began at 10 µM DMT. This discrepancy could simply be due to the difference between the in vivo application of Strassmann et al. and our in vitro application. Consequently, it can be assumed that heart rate and blood pressure are more sensitively affected by a combination of neuronal, vascular and cardiac effects of DMT after intravenous administration. In our experiments, only the direct effects on cardiac myocytes are responsible for any changes in force of contraction (or beating rate). Similarly, when ayahuasca preparations from the Amazon Basin were taken by human volunteers, heart rate and blood pressure augmented^32^. Further it was shown that DMT binds to 5-HT_1A,1B,1D_, and 5-HT_2A,2B,2C,6 and 7_ receptors^31,36^. However, 5-MeO-DMT binds with a high affinity to 5-HT_1A,1B,1D_, and 5-HT_5A,6 and 7_ receptors, but with a markedly less affinity to 5-HT_2A,2B, and 2C_ receptors compared to DMT^37^. Unfortunately, the binding affinities of DMT and 5-MeO-DMT to 5-HT_4_ receptors are not known, and the investigation of these parameters was beyond the scope of our study. Therefore, we could only compare the functional effects of DMT, 5-MeO-DMT and 5-HT between 5-HT_4_-TG and WT mice.

The present study demonstrated that DMT and 5-MeO-DMT exerted a concentration-dependent positive inotropic effect, and were less potent than 5-HT. Thus, we present data that 5-MeO-DMT and especially DMT, similar to cisapride, are partial agonists on 5-HT_4_ receptors, but also noted that the kinetic seems different because it took more time to reach a plateau than 5-HT in 5-HT_4_-TG.

Moreover, we could show that DMT and in particular 5-MeO-DMT can raise the phosphorylation state of phospholamban. This increased phosphorylation state of phospholamban may mediate the contractile effect of 5-MeO-DMT. Any augmented phosphorylation of phospholamban will lead to less inhibition of the Ca^2+^ pump (SERCA): SERCA would be pumping faster. This would be expected to lead to a more rapid relaxation of the left atrial preparations from 5-HT_4_-TG mice but not WT mice. Serotonin elevated the phosphorylation state of phospholamban in cardiac preparations from of 5HT_4_-TG^12^. Likewise, serotonin augmented the phosphorylation state of phospholamban in isolated atrial samples from patients^19^.

It should be noted, however, that there are some limitations of the study: For example, it is debatable whether the results obtained in mouse atria can be extrapolated to humans. The receptor density can be assumed to be different between 5-HT_4_-TG mice and humans and, furthermore, it is not clear if the cellular localization or signal transduction of the transgenic receptor is exactly the same as in human cardiomyocytes. However, this transgenic model has been successfully used several times to analyze cardiac effects of 5-HT and drugs or approved medications acting via 5-HT_4_ receptors in comparison to human atria (bufotenin^38^; psilocybin^39^; prucalopride and cisapride^40^). Another limitation is that we were not able examine the EC_50_ values of DMT and 5-MeO-DMT neither in our mouse model nor in human preparations. This would have been an opportunity to compare the effects of different drugs on the cardiac 5-HT_4_ receptor, but now we can only estimate this. On the other hand, this could mean that at concentrations that induce effects in the central nervous system, cardiac side effects may be unlikely.

In summary, our findings indicate that the hallucinogenic drugs DMT and 5-MeO-DMT can have cardiac side effects via human 5-HT_4_ receptors at least under certain circumstances, such as an overdose of DMT or 5-MeO-DMT. This knowledge might become important once DMT and 5-MeO-DMT are described to treat depression.

## Electronic supplementary material

Below is the link to the electronic supplementary material.

Supplementary Material 1

## Data Availability

The data of this study are available from the corresponding author upon reasonable request.
