# Supplementary material for: Cardiac effects of two hallucinogenic natural products, N,N-dimethyl-tryptamine and 5-methoxy-N,N-dimethyl-tryptamine
Source: Sci Rep. 2025 Feb 25;15:6715. doi: 10.1038/s41598-025-91400-6 (PMC11862204; doi:10.1038/s41598-025-91400-6)
Supplement: Supplementary file 1 — Supplementary Material 1 [file 41598_2025_91400_MOESM1_ESM.pdf]

5-MeO-DMT (for quantification)

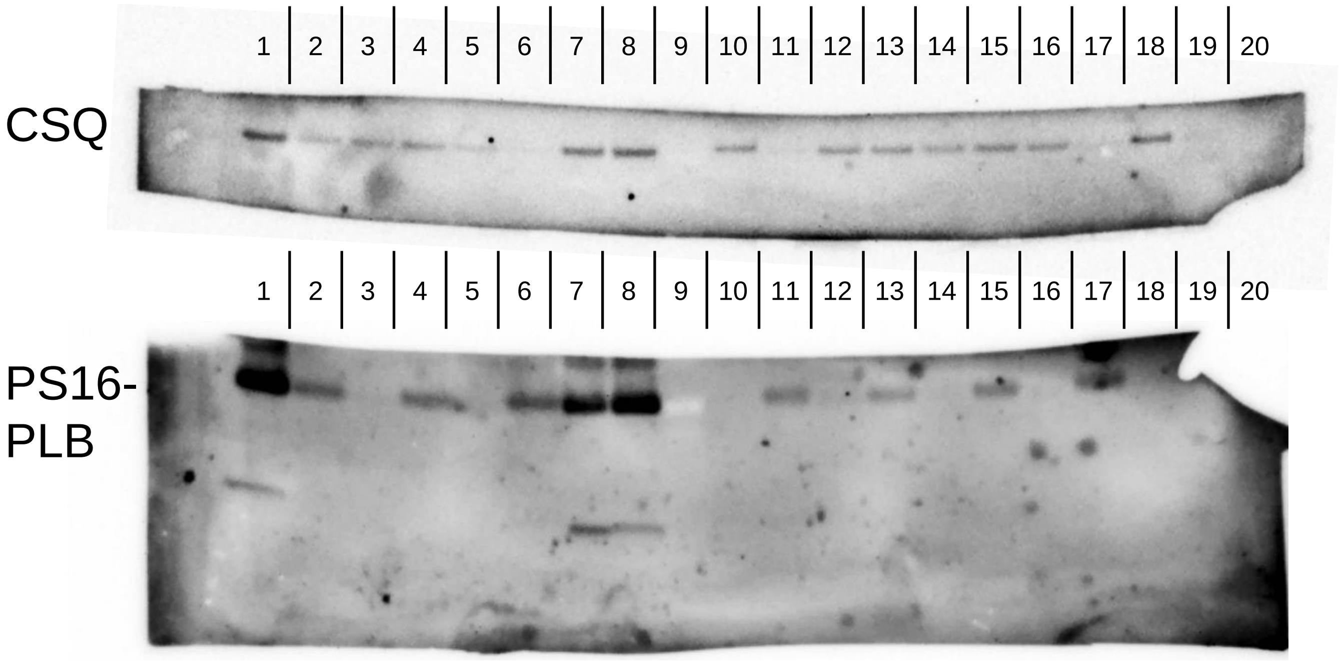

Legend:

1. Isoprenaline (positive control)
2. 5-HT$_4$-TG left atrium
3. WT left atrium
4. 5-HT$_4$-TG right atrium
5. WT right atrium
6. 5-HT$_4$-TG left atrium
7. Isoprenaline (positive control) boiled
8. Isoprenaline (positive control)
9. Rainbow marker
10. WT left atrium
11. 5-HT$_4$-TG right atrium
12. WT right atrium
13. 5-HT$_4$-TG left atrium
14. WT left atrium
15. 5-HT$_4$-TG right atrium
16. WT right atrium
17. 5-HT$_4$-TG left atrium
18. WT left atrium
19. 5-HT$_4$-TG right atrium
20. \- empty

DMT (for quantification)

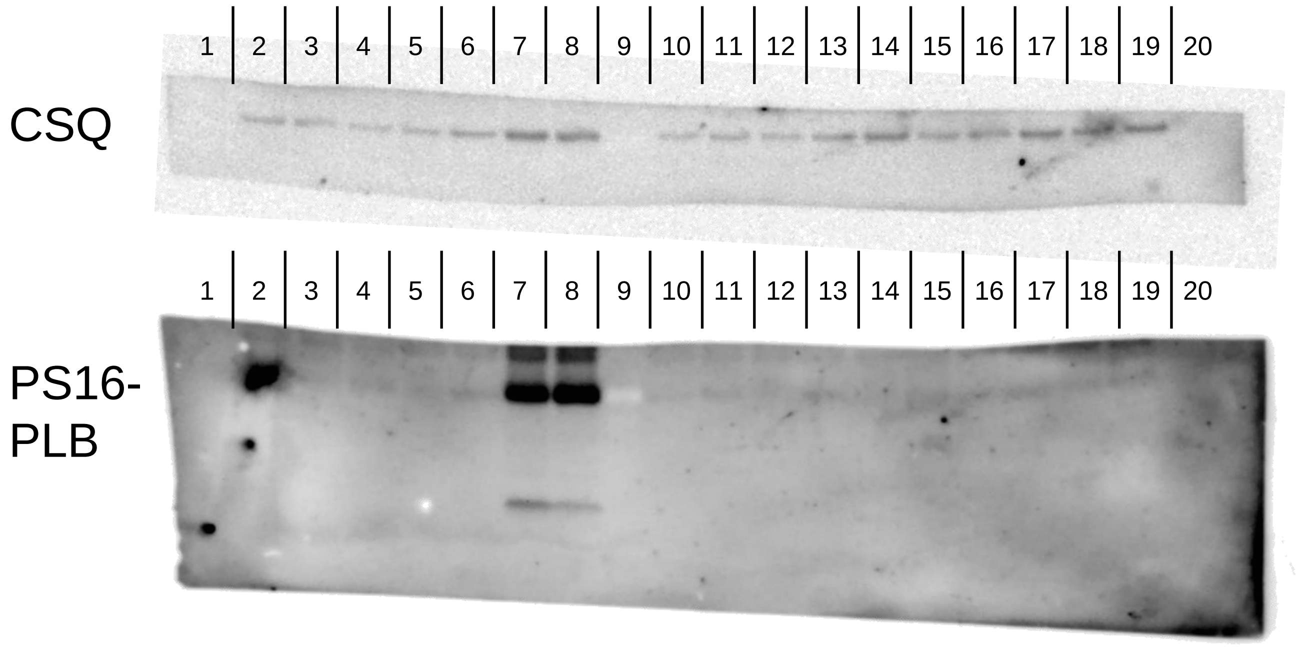

Legend:

1. - empty
2. 5-HT_4-TG left atrium
3. WT left atrium
4. 5-HT_4-TG right atrium
5. WT right atrium
6. 5-HT_4-TG left atrium
7. Isoprenaline (positive control) boiled
8. Isoprenaline (positive control)
9. Rainbow marker
10. WT left atrium
11. 5-HT_4-TG right atrium
12. WT right atrium
13. 5-HT_4-TG left atrium
14. WT left atrium
15. 5-HT_4-TG right atrium
16. WT right atrium
17. 5-HT_4-TG left atrium
18. WT left atrium
19. 5-HT_4-TG right atrium
20. - empty

(for presentation)

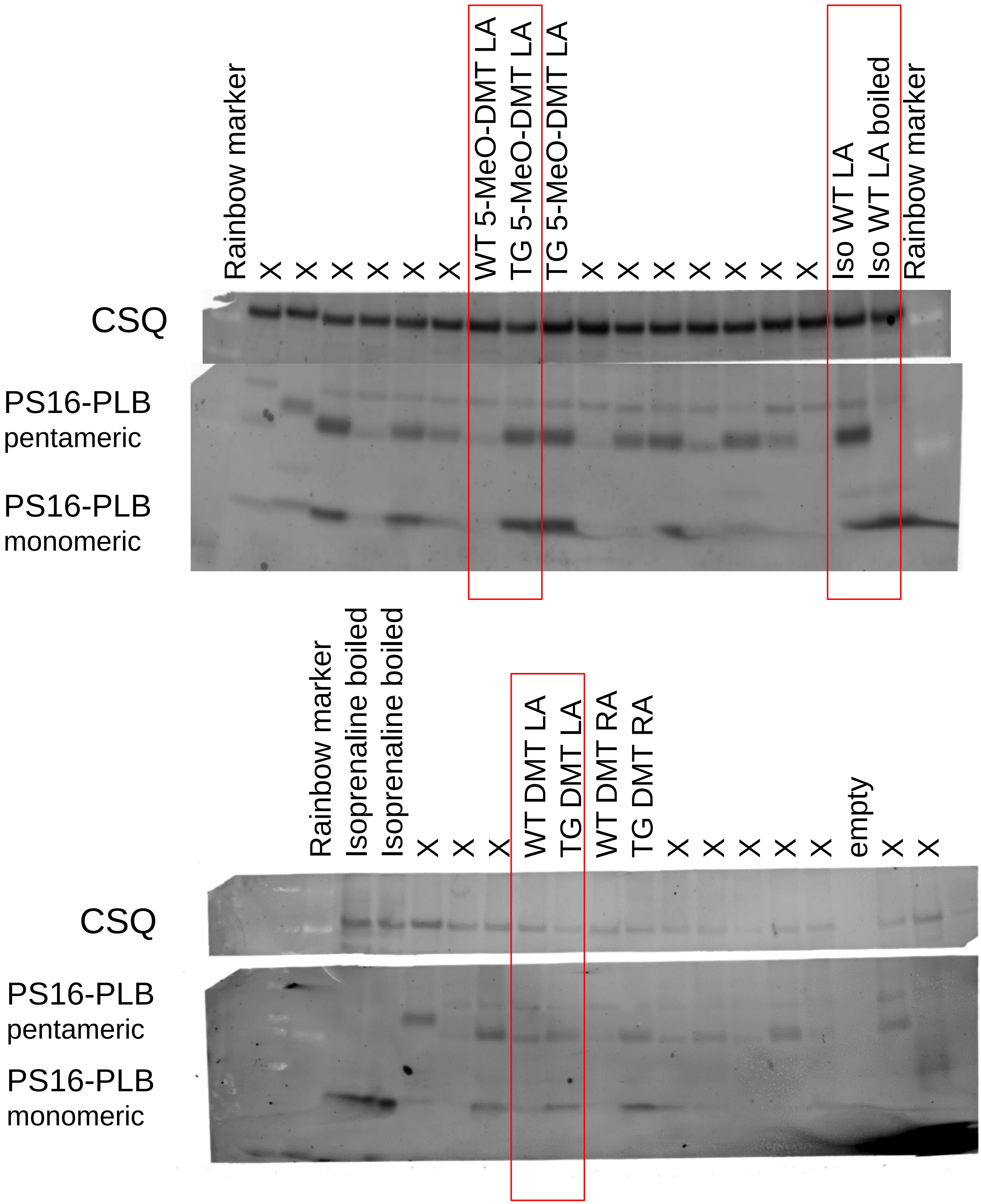

X = other samples not belonging to this project
